# Physiotherapists’ opinions, barriers, and enablers to providing evidence-based care: a mixed-methods study

**DOI:** 10.1186/s12913-022-08741-5

**Published:** 2022-11-21

**Authors:** Connor Gleadhill, Katarzyna Bolsewicz, Simon R. E. Davidson, Steven J. Kamper, Amanda Tutty, Emma Robson, Priscilla Viana Da Silva, Bruce Donald, Katherine Dooley, Joshua Manvell, Nicole Manvell, Andrew Delbridge, Christopher M. Williams

**Affiliations:** 1 School of Medicine and Public Health, University of Newcastle, Newcastle, Australia; 2 Hunter New England Population Health, Hunter New England Local Health District, Newcastle, Australia; 3 National Centre for Immunisation Research and Surveillance, Kids Research, Sydney Children’s Hospitals Network, Westmead, Australia; 4 School of Health Sciences, University of Sydney, Sydney, Australia; 5 Nepean Blue Mountains Local Health District, Nepean Hospital, Derby Street, Penrith, Australia; 6 Northern NSW Local Health District, Lismore, Australia; 7 Hunter New England Population Health, Wallsend, Australia; 8 John Hunter Hospital Physiotherapy, Hunter New England Local Health District, New Lambton, Australia; 9 School of Allied Health, Exercise and Sport, Charles Sturt University, Orange, Australia; 10 Department of Orthopaedic Surgery, John Hunter Hospital, Hunter New England Local Health District, New Lambton, Australia; 11 NuMoves Physiotherapy, Newcastle, Australia; 12 Regent Street Physiotherapy, New Lambton, Australia; 13 Mid North Coast Local Health District, Port Macquarie, Australia

**Keywords:** Physiotherapy, Evidence-based practice, Implementation, Mixed-methods, Evidence uptake, Research engagement

## Abstract

**Background:**

Physiotherapists deliver evidence-based guideline recommended treatments only half of the time to patients with musculoskeletal conditions. Physiotherapists’ behaviour in clinical practice are influenced by many cognitive, social, and environmental factors including time and financial pressures. Many initiatives aimed at improving physiotherapists’ uptake of evidence-based care have failed to appreciate the context involved in clinical decisions and clinical practice. Therefore, we aimed to describe: i) opinions toward evidence; ii) how evidence is accessed; iii) factors influencing evidence access; iv) factors influencing evidence application, for physiotherapists working in regional areas.

**Methods:**

We used a mixed-methods study with online survey and focus groups. We included registered physiotherapists in the survey and physiotherapists practising in regional New South Wales in the focus groups. Quantitative and qualitative data were used to inform all research objectives. We used eight domains of the Transtheoretical Domains Framework to design survey questions. We analysed quantitative and qualitative data in parallel, then integrated both sources through by developing a matrix while considering the Transtheoretical Domains Framework domains to generate themes.

**Results:**

Fifty-seven physiotherapists participated in the study (survey only *n* = 41; focus group only *n* = 8; both survey and focus group *n* = 8). Participants reported that evidence was important, but they also considered patient expectations, colleagues’ treatment choices, and business demands in clinical decision making. Physiotherapists reported they access evidence on average 30 minutes or less per week. Competing demands like business administration tasks are barriers to accessing evidence. Participants reported that patient expectations were a major barrier to applying evidence in practice. Environmental and systemic factors, like funding structures or incentives for evidence-based care, and social factors, like lacking or having a culture of accountability and mentorship, were reported as both barriers and enablers to evidence application.

**Conclusions:**

This study provides context to physiotherapists’ opinion, access, and application of evidence in clinical practice. Physiotherapists’ provision of evidence-based care may be improved by enhancing structural support from workplaces to access and apply evidence and exploring discrepancies between physiotherapists’ perceptions of patient expectations and actual patient expectations.

**Supplementary Information:**

The online version contains supplementary material available at 10.1186/s12913-022-08741-5.

## Contributions to the literature

- Contextual factors that impact evidence-based care provision are appreciated but rarely systematically assessed in implementation research.
- Although physiotherapists consider evidence important, clinicians must contend with multiple contextual factors when making clinical decisions and accessing and applying evidence in practice, for example business demands and patient expectations.
- While contextual factors impacting evidence access and application may often be difficult to alter, we discuss potentially modifiable targets for future strategies to improve evidence-based care provision.

## Introduction

Physiotherapists deliver guideline recommended treatments only half of the time to patients with musculoskeletal conditions [1]. Previous research has identified barriers that impact physiotherapists’ ability to put evidence into practice [2–11]. Research is commonly generated without addressing clinical priorities [12–15]. Evidence often lacks vital information about treatment parameters [16, 17]. Physiotherapists also experience challenges when trying to implement evidence in practice [18–20]. For example, physiotherapists may lack the resources to access evidence behind paywalls [3, 4], and have low confidence to appraise statistics [5].

Physiotherapists’ behaviour in evidence-based practice is influenced by a host of contextual factors, such as workplace resources or organisational culture [21–23]. Improving the uptake of evidence into practice involves studying health professional behaviour and then targeting specific factors influencing these behaviours with strategies (called implementation strategies) [19, 20, 24]. Studies that evaluate implementation strategies among physiotherapists rarely consider broader contextual factors [25, 26]. Contextual barriers to care provision differ depending on the practice setting [24]. For example, learning opportunities and supervision may not be as readily available in regional areas versus capital cities [27, 28]. Studies investigating barriers to evidence-based practice often sample from an ill-defined population [2–5]. To improve the uptake of evidence into practice, studies investigating specific contextual factors that impact isolated behaviours are needed [19–26].

### Objectives

Our objectives were as follows:

I Describe the opinions of physiotherapists working in regional areas toward evidence in relation to their clinical decision making.
II Describe how physiotherapists working in regional areas access evidence.
III Describe the factors influencing physiotherapists working in regional areas evidence access.
IV Describe the factors influencing physiotherapists working in regional areas evidence application.

## Methods

### Design

This study was embedded into the establishment of a practice-based research network for physiotherapists in regional Australia, which aims to co-produce research evidence and improve the local uptake of evidence-based care for musculoskeletal conditions. To address research objectives, we used a mixed methods design involving online survey and online focus groups. Quantitative and qualitative data from survey responses were used to inform all research objectives. We wanted to quantitatively describe contextual factors involved in clinical decision making (objective one), patterns of evidence access (objective two), and Theoretical Domains Framework (TDF) constructs (version one) involved in accessing and applying evidence (objectives three and four) [20]. The TDF offers a theoretical lens through which to view the cognitive, affective, social and environmental influences on behaviour, and has been extensively used in implementation research [20, 29, 30]. We used qualitative data to explore all four research objectives in more depth [31, 32]. We considered focus groups ideal to allow participants to share ideas on the challenges to evidence access and application [32–34]. The TDF was also used as a framework for data analysis and interpretation [20]. We refer to the process of generating research as ‘research’, and the end-product as ‘evidence’ throughout [35].

### Participants - survey

We were interested in the context surrounding physiotherapists practising in regional Australia (see footnote^1^ for more details) [36]. For the online survey, we chose a pragmatic recruitment strategy to maximise response rate. We invited physiotherapists through public social media posts inviting regional physiotherapists to answer our survey. Posts were placed on personal pages and in discussion groups for allied health practitioners.

We included participants if they were registered physiotherapists and excluded participants if they were any other health professionals.

### Participants - focus groups

For the focus groups, we purposively sampled from three groups of physiotherapists to enable a more rounded discussion of the challenges with evidence-based care provision: those working in private clinics; those working in the public health system; and physiotherapy researchers [37]. We invited physiotherapists who practised in regional New South Wales (NSW) via individual email [36]. Emails were sent to existing connections of the author team.

We included participants if they were registered physiotherapists working in regional NSW and excluded physiotherapists who did not work in regional NSW.

### Outcome measures

The survey consisted of five sections (see Appendix 1).

- Demographics: age; experience level (years); location of practice; clinical area [38].
- Opinions about evidence in relation to clinical decision making.
- Amount of time per week participants reported accessing evidence, in various mediums.
- Barriers to evidence access and application. This was assessed using 12 five-item Likert scale questions, each assessing agreement to the following statement, ‘It’s hard to apply evidence because, … ’. Here, we listed TDF domains (knowledge; skills; beliefs about capabilities; motivation and goals; environmental context and resources; social influences; emotion, and the nature of behaviours). Each of the 12 TDF domains includes a number of constructs, which researchers can include in qualitative or quantitative assessment health professional behaviour [20]. We used eight TDF domains and selected one or two key constructs from each domain after careful consideration of authors’ lived experience of the behaviours under investigation and prior data on physiotherapists’ barriers and enablers to evidence-based care provision [2–11].
- Enablers to evidence access and application. Participants were asked to rate four enablers on a five-item Likert scale.

We used interview questions to explore more TDF domains and constructs (online supplement 1). We elicited participants’ opinions on where evidence fits into providing care, perceived barriers to accessing and applying evidence, and suggestions to address these barriers. We kept the interview guide flexible, to follow lines of discussion as they came up [39].

Quantitative data were obtained from online survey and qualitative data were obtained from online focus groups and two free-text responses in the survey. Survey responses were captured between 22 July and 29 August 2020, using Research Electronic Data Capture (REDCap) database [40]. Online focus groups were held between 29 July and 6 August 2020. Each focus group lasted approximately 60 minutes and was recorded using videoconferencing software Zoom® [41]. Focus groups had a maximum of eight participants and two researchers.

### Data analysis

Quantitative and qualitative data were organised separately before we used a mixed-methods analysis to develop themes. We organised and described quantitative survey data as either means and standard deviations or frequency counts and proportions of the total sample. As this was an exploratory study, we did not perform a power calculation for quantitative data.

CG transcribed and cleaned audio files from the focus groups and then de-identified and organised data in Nvivo 12 [42]. Two researchers (CG and SD) coded focus group data for key concepts and ideas simultaneously (SD coded data using a more inductive process, having not been involved in the development of survey questions) [43]. To achieve a mixed-method analysis, we followed three stages.

First, we created a ‘baseline’ matrix for each research objective [44, 45]. The matrix had one column for quantitative support and one for qualitative support. For research objectives 3 and 4, CG pre-populated the matrix rows with all 12 TDF domains [44, 45]. CG then consulted qualitative and quantitative data and filled in each cell according to the level of support from each source [46, 47]. Where there was no qualitative or quantitative support for a TDF domain, this row was removed from the ‘baseline’ matrix [20, 46–48].

Second, CG created an ‘expanded’ matrix by adding extra rows unrelated to TDF domains, where there was agreement between qualitative data categories (groups of similar codes) and quantitative data [46–48]. CG then explored the level of support, congruence, or dissonance in data sources across matrix rows, and the relationships between TDF domains to create themes [45–48]. We used this ‘expanded’ matrix (with included themes) in the following stage of analysis.

Third, CG, SD, KB, and CW refined the theme matrix by discussing the relationships between themes [46–50]. We removed themes with least support, included themes with disagreement between data sources, and merged similar themes [46–50]. This process resulted in a ‘final’ matrix of themes (online supplement 2).

Following our mixed-methods analysis, we determined any differences between regional physiotherapists’ survey data and all survey responses by performing a subgroup analysis of regional physiotherapists’ data only.

Both CG and SD, who coded data, are physiotherapists with lived experience of the challenges to accessing and applying evidence in practice. CG has professional relationships with many focus group participants. This shaped data collection, analysis, and theory development.

## Results

### Flow of participants through the study

The total sample consisted of 57 physiotherapists (survey only *n* = 41; focus group only *n* = 8; both survey and focus group *n* = 8) who ranged in clinical experience, predominantly practised in regional Australia (*n* = 42, 74%), and had a musculoskeletal clinical focus (*n* = 33, 57%) (Table 1). Partially completed surveys (*n* = 1) were included in the analysis. Due to our recruitment methods for the online survey, a response rate cannot be calculated (a voluntary survey link advertised on social media). All participants who were invited to focus groups took part.

**Table 1:** Demographics of physiotherapists who were included in the mixed methods study (*n* = 57). Eight participants took part in both the survey and focus group

	Survey	Focus Groups	Total sample
	*n* = 49	*n* = 16	*n* = 57
**Age** (years) *mean (SD)*	38 (27 - 49)	41 (33-49)	40 (29-51)
**Years in practice** (n (%))			
1-5	10 (20%)	5 (16.5%)	13 (23%)
6-10	16 (33%)	3 (28%)	13 (23%)
11-15	6 (12%)	2 (16.5%)	8 (14%)
16-20	6 (12%)	5 (11%)	10 (17%)
21+	11 (23%)	1 (28%)	13 (23%)
**Location of practice** (n (%))			
Regional	35 (72%)	15 (89%)	42 (74%)
Rural	3 (6%)	0 (0%)	3 (5%)
Remote	1 (2%)	1 (5.5%)	2 (3.5%)
Don’t know	1 (2%)	0 (0%)	1 (2%)
I practice outside of Australia	9 (18%)	0 (0%)	9 (15.5%)
**Clinical area of practice** (n (%))			
Cardiothoracics	1 (2%)	0 (0%)	1 (2%)
Chronic pain	5 (10%)	1 (6%)	5 (8.5%)
Chronic respiratory disease	1 (2%)	1 (6%)	2 (3.5%)
Continence and Women’s Health	1 (2%)	0 (0%)	1 (2%)
Gerontology	1 (2%)	0 (0%)	1 (2%)
Musculoskeletal	29 (60%)	11 (69%)	33 (57%)
Neurology	0 (0%)	1 (6%)	1 (2%)
Oncology	1 (2%)	0 (0%)	1 (2%)
Orthopaedics	3 (6%)	0 (0%)	3 (5%)
Sports	6 (12%)	2 (13%)	8 (14%)
Whiplash	1 (2%)	0 (0%)	1 (2%)

### Findings

The complete results of survey data can be found in Appendix 1. The results of the subgroup analysis of regional physiotherapists’ survey data (*n* = 35, online supplement 2) did not significantly alter the proportions in response categories within included themes. We have structured results by research objectives, and key themes for research objective one and two. For research objectives three and four, we have structured results by TDF domains.

#### Research objective 1: opinions towards evidence in relation to clinical decision making

For complete results of mixed methods analysis for objective one see online supplement 2.

#### Positive attitude

Nearly all survey participants (96%) considered evidence as either important or very important in clinical decision making. Most survey participants (86%) also agreed that evidence informs their treatment choice. For example, one participant described:

*“[Evidence is] Crucial to pushing the profession forward.”* (Participant 1, focus group 1).

#### Integration of many factors

Participants reported it is important to consider many other factors when using evidence in clinical decision making. Patient expectations were either important or very important when making clinical decisions for most survey participants (90%). 77% of survey participants rated colleagues’ choices as important or very important and participants reported the clinical environment can shape clinical decisions.

#### Tension between factors involved in clinical decisions

Participants reported that other considerations of clinical practice, like experience and business demands may sometimes conflict with evidence. For example:

*“As far as delivering high quality care, I have always struggled with that one. I feel like it’s really hard to give a good treatment in a short period of time that’s financially viable that a person is willing to pay for.”* (Participant 15, focus group 4).

#### Research objective two: patterns of accessing evidence

Quantitative results for objective two are provided in Table 2. Online supplement 2 summarises the results of the mixed-methods analysis.

**Table 2:** How do physiotherapists access evidence? Quantitative results (*n* = 49)

‘How much time do you spend accessing evidence in the below mediums:’	< 10 minutes per week	10-30 minutes per week	30-60 minutes per week	1-2 hours per week	> 2 hours per week
Evidence summaries	8 (16%)	18 (37%)	8 (16%)	11 (23%)	4 (8%)
Listening to podcasts	18 (37%)	10 (20%)	9 (19%)	7 (14%)	5 (10%)
Article abstracts	12 (25%)	21 (43%)	10 (20%)	2 (4%)	4 (8%)
Full text articles	14 (29%)	15 (31%)	7 (14%)	6 (12%)	7 (14%)
Reading blogs	13 (27%)	20 (41%)	8 (16%)	6 (12%)	2 (4%)

Physiotherapists dedicate a small amount of time to accessing evidence, using multiple different mediums. Over 50% of survey participants reported spending 30 minutes or less per week on accessing evidence, across all mediums.

Participants reported engaging in quality assessment (triaging) when accessing evidence, but it is unclear what such quality assessment entails. For example*:*

*“If any physio related stuff comes up, I’ll usually notice it and triage if I’m actually going to read it or not.”* (Participant 1, focus group 1).

#### Research objective three: factors influencing physiotherapists’ evidence access

We report the results of research objective three by TDF domains. Table 3 presents themes on barriers and enablers to accessing evidence.

**Table 3:** Understanding the barriers and enablers of regional physiotherapists in evidence access (*n* = 57)

TDF Domain (construct)	Quantitative data	Qualitative data	Theme
Knowledge	**Participants disagreed this was a barrier** - 84% of participants either disagreed or strongly disagreed with the statement, “It’s hard to apply evidence because I don’t know where to find evidence.”	**Participants disagreed this was a barrier** ***Focus group data:*** *“The evidence is out there, and people have access to it.”* *“But now, it’s still somewhat easy peasy because they are reviewed, whether they be Cochrane reviewed or at least a systematic review.”*	***Prioritisation*** Access limitations driven by competing demands of clinical practice (and resulting time limitations) not the knowledge of how to access evidence.
Skills	**Participants disagreed this was a barrier** - 55% of participants either disagreed or strongly disagreed with the statement, “It’s hard to apply evidence because it’s hard to access evidence.”		
Context and resources (Environmental stressors)		**Reported as a barrier** ***Focus group data:*** *“The big problem is always having enough time to access evidence.”* ***Survey qualitative data:*** *“My only barrier is time... which is more of an excuse than a barrier.”* *“Not only are we trying to stay abreast of current evidence and trying to incorporate that into our practice. Most business owners, like myself, carry a bigger caseload than they should. And then with what time you have left over after doing all that, you’re trying to run a business. And so, you’ve got all of the financial aspects of that.”*	
Context and Resources (Resource availability)		**Reported barrier** ***Survey qualitative data:*** 5 free text responses to the question, “Please list any other barriers you have to accessing and applying evidence to your practice,” mentioned paywalls or restricted access to full text articles. For example, *“Access to full text journals has been main barrier.”*	***Paywalls*** Paywalls were viewed as a barrier to accessing evidence.
		**Reported enabler** ***Survey qualitative data:*** 2 free-text responses to the statement, ‘Please list anything else that might make it easier for you to access and apply research to your practice’ listed reduced cost or no paywalls. For example, *“Better access to full text articles without paywall barriers.”*	Removing paywalls was viewed as an enabler to accessing evidence
Social influences		**Reported enabler** ***Focus Group data:****“It [evidence] all comes through Facebook. I’ve always kind of got an eye on what’s gone on, because there’s business stuff gone on there and I’m not kind of just sitting there all day on it. But I’m always just – you know – having a quick look. So, yeah, if anything of that – any physio related stuff comes up, I’ll usually notice it and again triage if I’m going to actually read it or not.”*	***Social media***

#### Knowledge and skills

Most survey participants (84%) disagreed that it is ‘hard to find evidence’, while 55% disagreed that it is ‘hard to access evidence’. Participants expressed that they know how to access evidence, meaning the TDF domains of knowledge about skills, were not barriers. One participant reported:

“*The evidence is out there, and people have access to it.”* (Participant 10, focus group 3).

##### Environmental context and resources

Paywalls were reported as a barrier. However, participants also reported that removing paywalls would make it easier to access evidence.

Participants reported time as a barrier to accessing evidence, and this time limitation is driven by competing demands, for example, seeing patients or fulfilling business administration tasks. For example, one participant expressed difficulty in prioritising accessing evidence over other competing demands:

“*Not only are we trying to stay abreast of current evidence and trying to incorporate that into our practice. Most business owners, like myself, carry a bigger caseload than they should. And then with what time you have left over after doing all that, you’re trying to run a business.”* (Participant 7, focus group 3).

##### Social influences

Participants also reported that using social media facilitates evidence access, as they frequently use social media for other reasons like connecting with friends.

#### Research objective four: barriers and enablers in applying evidence

We report the results of research objective four by TDF domains. Table 4 presents themes on barriers and enablers to applying evidence.

**Table 4:** Understanding the barriers and enablers of regional physiotherapists in evidence application

TDF domain (construct)	Quantitative data	Qualitative data	Theme
Skills	**Not reported as a barrier** - 51% either disagreed or strongly disagreed with the statement, “It’s hard to apply evidence because It’s hard to know what good quality research evidence is.”	**Reported barrier** *“There’s so much noise coming from all of the research that happens, both good quality and certainly less quality, that it is hard to see how that fits in with the clinical practice guidelines for treatment of different conditions.”*	**Volume of evidence** Critical appraisal is difficult in the context of an ever-increasing amount of evidence.
		*“Unfortunately, the thing about evidence is it’s always changing and so keeping up with that, then you know, you get people to follow one thing and then a few years down the line you tell them something else.”*	Keeping up to date with changing recommendations is difficult*.*
Beliefs about capabilities (perceived behavioural control)	**Reported barrier** - 60% either agreed or strongly agreed to the statement, “It’s hard to apply research evidence because my patients expect certain treatments that aren’t evidence based.”	**Reported barrier** *“The patient can often be very confused around what to believe or pursue for treatment. So often trying to bring that back into some sort of order and structure for creating a management plan for them can be a bit challenging if they come with a lot of those pre-conceived ideas of what they think they need or what they’ve been told with good intention from others.”*	**Patient expectations** Patients may expect non-evidence-based treatment
Beliefs about consequences		**Reported barrier** *“I think inevitably we’ll have to deal with it. Because you’ve got the patient who comes with those preconceived beliefs and past experiences and current situation that, again if you’re not building that rapport and trust initially, everything else you do isn’t gunna [going to] be taken on board.”*	Choosing one treatment over another may have consequences on the therapeutic alliance.
Environmental context and resources		**Reported barrier and enabler** **Barrier:** *“But right now, it doesn’t work like that. So, we have the clinicians who choose to deliver largely what they want and if it’s loosely physiotherapy, most of it is paid for.”*	**System factors** Lack of incentive to provide evidence-based care
		**Enabler:** *“If you wanna [want to] get at this issue then it needs to be addressed, um, we need to be putting the effort in so the information to the payers of healthcare, so that they demand high value care or only pay for high value care”*	Funding evidence-based care would enable better evidence-based care provision
		*“And I don’t know how you can stamp out that practice without some sort of accountability structure on a more systemic level.”* *“I work at a multidisciplinary clinic. And talking to the doctors. They’re audited very heavily by Medicare and every practice, every investigation, every diagnosis they make, even down to the amount of minutes that they spend in the consult is all audited by Medicare. And that’s a systemic thing, that covers an auditor.”*	Creating a general system of accountability.
		*“But I wonder if it’s about how you can incentivise but create the structure around making the right thing to do easy.”* *“That is systemic that doesn’t require practitioners to have to consciously make a decision all the time to behave this way. That it’s built in to their, more than culture, the system somehow.” (In relation to Medical Officers)*	Systemic supports to make evidence-based care easier
Social influences	**Reported enabler** 86% either ‘agreed’ or ‘strongly agreed’ with the statement, “It would make it easier to apply research evidence if I were able to connect with other like-minded clinicians to discuss applying evidence in practice.”	**Reported barrier and enabler** **Barrier:** *“We are first contact practitioners, so we do have that responsibility and yet we don’t necessarily, whether it’s public or private, have the infrastructure and maybe the really deep-seeded culture of accountability.”*	**A culture of accountability** A lack of a culture of accountability is a barrier to evidence application
		**Enabler:** *“Like I think about it in relation to medicine and of course the stakes are so completely different in medicine as well, in terms of saving lives and they have much more ability to cause harm. But it’s really in bred, much more, in them about that accountability for their practice maybe.”*	A culture of accountability, which may be more present in other professional cultures, may enable evidence application.
		**Barrier:** *“If bosses are pretty set in their ways, in – you know – using or not, maybe not staying up to date and then are educating new grads on traditional ways of physio, then – you know – it’s not quite likely that things are gunna [going to] move forwards.”*	**Mentorship** Senior physiotherapists, or supervisors, may make it harder to apply evidence in practice.
		**Enabler:** *“I’d like my staff to hear the same things that I would say from somebody else as well.”* *“But then of course with mentors or other clinicians to be able to discuss it and then learn how to put that into practice.”*	However, mentors can also enable the application of evidence in practice
Reported barriers and enablers that are not informed by the TDF			
	**Reported barrier and enabler** **Barrier:** - 65% of participants either agreed (47%) or strongly agreed to the statement, “It’s hard to apply evidence because a lot of evidence doesn’t answer my clinical problems.” 29% of participants either disagreed or strongly disagreed with the statement.	**Reported barrier and enabler** **Barrier:** *“Researchers are really good at asking questions, but sometimes the questions aren’t that relevant for us.”*	***Research relevance*** Clinical relevance issues
		*“The other area is that it’s not always relevant to the population that you’re dealing with in the clinic. Whether it’s studies on knees or hamstrings or whatever in elite athletes and I’m trying to treat a social soccer player or something like that. Or someone who’s workin’ full time and got kids and hasn’t got time to spend every day in the gym or do these certain things or like return to running programs and like that sort of stuff.”*	Generalisability issues
		*“There’s some really good, high-quality research out there but you can’t implement it, because it’s so controlled and it’s so sterile and it’s got nothing to do with what we do in our clinic or practice.”*	Implementability issues
	**Enabler:** - 78% either ‘agreed’ or ‘strongly agreed’ with the statement, “It would make it easier to apply research evidence if I get support to answer questions relevant to my patients/clinic.”	**Enabler:** *“Like I think if there were a much closer relationships between the people who are doin’ the research and the people who are running the clinics, then researchers are asking more relevant questions to clinical life.”* *“I sort of need researchers to better understand what clinic life is like. So that they’re asking better questions.”*	Making research questions more relevant

##### Skills

Skills were a barrier to research application; participants reported that the skill of critical appraisal is difficult in the context of the ever-increasing amount of evidence.

##### Beliefs about capabilities and beliefs about consequence of behaviour

Patient expectations were a barrier to research application in two ways. 60% of survey participants agreed to the statement, “it’s hard to incorporate evidence into practice because patients expect certain treatments that aren’t evidenced based”. Participants reported that perceived impacts on the therapeutic alliance is a barrier to applying evidence. They viewed that the consequences of choosing one treatment over another may have negative effects on the therapeutic alliance between clinician and patient. For example:

*“Again, if you’re not building that rapport and trust initially, everything else you do isn’t gunna* [going to] *be taken on board.”* (Participant 10, focus group 3).

##### Environmental context and resources

System factors were reported as both barriers and enablers to evidence application. Participants reported healthcare funders often do not require care to be evidence-based, therefore physiotherapists experience a lack of financial incentive to apply evidence in practice. For example:

*“But right now, it doesn’t work like that. So, we have the clinicians who choose to deliver largely what they want and if it’s loosely physiotherapy, most of it is paid for.”* (Participant 5, focus group 2).

Consequently, participants suggested funding structures supportive of evidence-based care would encourage physiotherapists to apply evidence in their clinical practice. For example:

*“And I don’t know how you can stamp out that practice without some sort of accountability structure on a more systemic level.”* (Participant 10, focus group 3).

Participants also reported system factors could function as an enabler to evidence application by facilitating clinical decision making to preference evidence-based options, rather than non-evidence-based options. For example:

*“I wonder if it’s about how you can incentivise but create the structure around making the right thing to do easy.”* And *“That is systemic that doesn’t require practitioners to have to consciously make a decision all the time to behave this way. That it’s built in to their, more than culture, the system somehow.”* (Participant 8, focus group 3).

##### Social factors

Participants noted several factors in their social environment that either facilitated or hampered applying evidence in clinical practice. Participants reported that a lack of professional culture towards accountability to evidence-based care standards may be a problem, and consequently, developing such cultures may enable better application of evidence in practice.

Participants also reported that mentorship can be both a barrier and an enabler to evidence application. For example, one participant expressed the downside of poor mentorship:

*“If bosses are pretty set in their ways, maybe not staying up to date, and are educating new grads on traditional ways of physio, then it’s not likely that things are going to move forwards.”* (Participant 14, focus group 4).

However, 86% of survey participants agreed with the statement, “It would make it easier to apply research evidence if I were able to connect with other like-minded clinicians to discuss applying evidence in practice.”

##### Research relevance

Participants questioned research relevance to their practice. 65% of survey participants agreed with the statement, ‘it’s hard to apply research evidence because research doesn’t answer my clinical problems’. For example, one participant reported:

*“Researchers are really good at asking questions, but sometimes the questions aren’t that relevant for us.”* (Participant 16, focus group 4).

Participants also reported that evidence may not be implementable in clinical practice. For example:

*“There’s some really good, high-quality research out there but you can’t implement it, because it’s so controlled and it’s so sterile and it’s got nothing to do with what we do in our clinic or practice.”* (Participant 6, focus group 2).

Most participants noted that improving evidence relevance would be helpful. 78% of survey participants agreed that it would be easier to apply evidence if they were supported to answer questions relevant to their patients or clinic. For example:

*“I sort of need researchers to better understand what clinic life is like. So that they’re asking better questions.”* (Participant 16, focus group 4).

## Discussion

In our study, participants reported that evidence is important when making clinical decisions, but it is only one factor among many to consider in clinical practice. Participants report dedicating small amounts of time weekly to accessing evidence across different kinds of mediums. Participants indicated addressing contextual barriers would ease evidence application and suggested removing paywalls, better mentorship, funding of evidence-based care, and establishing a culture of accountability within the profession may result in improvements.

### Strengths

We have defined and explored two specific behaviours in our study; evidence access and application. Most studies fail to isolate specific behaviours involved in evidence implementation, instead referring to a confusing array of terms and behaviours [2–11]. We have also applied a well-established framework, the TDF [20]. To our knowledge, this is the first study to quantify physiotherapists patterns of evidence access across multiple mediums (like blog posts, podcasts, and full text articles).

### Limitations

The focus groups had a dual focus; to inform the research objectives and inform the establishment of a practice-based research network for physiotherapists. The network had the explicit purpose of co-designing and implementing more clinically relevant evidence. This likely altered participants’ perspectives towards evidence relevance as both a barrier and enabler to evidence application. Participants held a highly positive opinion of evidence, which may not be representative of the profession more generally. However, this limitation is likely a feature of most qualitative research investigating physiotherapists’ opinion of evidence [51–53]. Our survey recruitment strategy led to responses from physiotherapists who practice outside Australia (we used public social media posts to maximise recruitment). However, our subgroup analysis demonstrated this may not have impacted themes generated through mixed-methods analysis.

### Implications for modifiable contextual factors

Although our study demonstrates many factors can be both barriers and enablers to research access and application, we will limit our discussion to potentially modifiable contextual factors.

Social factors are a well-recognised modifiable factor to improve the uptake of evidence into practice [20–25]. Our results reinforce the importance of peer-to-peer learning and mentorship to improve evidence uptake in practice [26]. Empirical evidence suggests clinicians in regional and rural areas may not have equal access to mentorship opportunities than their metropolitan counterparts [27, 28]. Rather than lacking access to mentorship, participants in our study reported that poor, outdated mentorship was a barrier to applying evidence in practice. Due to the scope of our study (regional physiotherapists), we have not captured social barriers faced by rural clinicians. We recommend future research targets rural and remote settings to better assess the specific barriers faced in more isolated clinical contexts.

More effort is needed to understand how physiotherapists prioritise evidence access among the competing demands of clinical practice. Participants consistently report time pressures as a barrier to evidence uptake and implementation [2, 8–11]. However, clinicians often express being ‘time poor’, without also voicing contextual and driving factors [8–10]. For example, clinicians may feel under stress from general workload pressure or leave the behaviour in question until last (after more pressing priorities) [8–10]. It would be unreasonable to assume that physiotherapists would prioritise accessing evidence above seeing patients and administration tasks like notetaking. Initiatives like evidence-based case discussions may ensure clinicians purposefully access and appraise evidence and lead to positive attitudes towards evidence-based practice [54, 55]. However, adding initiatives like case discussions onto an already busy clinical schedule may force the physiotherapist to juggle yet more competing demands. Such initiatives may not be sustainable or valuable to clinicians if they are not prioritised. It is first necessary to understand how, and under what circumstances, clinicians prioritise accessing evidence among competing demands (for example, through discrete choice experiments). Ultimately, initiatives may have to involve dedicated (paid) time to access evidence.

Participants in our study reported that patients may expect certain treatments that are not evidence based, which makes applying evidence difficult. Participants indicated they provide non-evidence-based care options to preserve the therapeutic alliance. The original description of evidence-based practice clearly outlines that clinicians should incorporate and respect patient preferences [56]. Although clinicians should respect patient preferences, evidence-based practice demands more of clinicians than simply meeting patient expectations [56, 57]. While there is some evidence that patients may hold expectations that do not align with evidence-based care [58], a causal link between patient expectations and care outcomes is far from clear [59, 60]. Patient outcomes are improved when patients attend a physiotherapist with good communication skills or receive a consultative care experience [61]. We recommend longitudinal studies provide clarity on any hypothetical causal relationship between expectations and care outcomes. Further research should first aim to understand whether clinician’s perceptions of patient expectations align with the patient’s actual expectations [62, 63].

Our results demonstrate that a lack of clinically relevant evidence is a significant barrier to applying evidence in practice. Irrelevant research can stem from researchers focusing on problems that are not significant problems for clinicians or including treatments that can’t be readily implemented in practice [13–15]. This disparity between what information clinicians need day-to-day and the information evidence provides has resulted in widespread policy push to ‘co-produce’ research [64–66]. We recommend that researchers collaborate with clinicians to ensure full contextual appreciation of the challenges of evidence-based care delivery and treatments included in research are ‘real-world’ implementable [64–68].

## Conclusions

This study places physiotherapists’ opinion, access, and application of evidence into the context of clinical practice. Although physiotherapists consider evidence important, it is one among many factors that must be considered when making clinical decisions. Competing demands of clinical practice, like business administration, must be prioritised alongside accessing evidence. Some contextual factors like system and structural factors, for example funding structures, may be difficult to alter. However, physiotherapists’ provision of evidence-based care may be improved by enhancing structural support from workplaces to access and apply evidence and exploring discrepancies between physiotherapists’ perceptions of patient expectations and actual patient expectations. Researchers and clinicians working collaboratively may ensure research answers more relevant questions and is more considerate of the context of clinical practice.

## Supplementary Information

**Additional file 1:** **Appendix 1.**

**Additional file 2.** Online supplement 1.

**Additional file 3.** Online supplement 2.

## Data Availability

The datasets during and/or analysed during the current study available from the corresponding author on reasonable request.

## Abbreviations

NSW: New South Wales
TDF: Theoretical Domains Framework
REDCap: Research Electronic Data Capture

## Footnotes

1 We have used Australian Bureau of Statistics (ABS) Greater Capital City Statistical Area (GCCSA) Structure to define ‘Regional Australia’. Anything outside of a defined Greater Capital City is considered as Regional Australia [36].

## References

[1] Zadro J, O’Keeffe M, Maher C (2019). Do physical therapists follow evidence-based guidelines when managing musculoskeletal conditions? Systematic review. BMJ Open.

[2] Scurlock-Evans L, Upton P, Upton D (2014). Evidence-based practice in physiotherapy: a systematic review of barriers, enablers and interventions. Physiotherapy.

[3] Salbach NM, Guilcher SJ, Jaglal SB (2010). Determinants of research use in clinical decision making among physical therapists providing services post-stroke: a cross-sectional study. Implement Sci.

[4] Kamwendo K (2002). What do Swedish physiotherapists feel about research? A survey of perceptions, attitudes, intentions and engagement. Physiother Res Int.

[5] Jette DU, Bacon K, Batty C (2003). Evidence-based practice: beliefs, attitudes, knowledge, and behaviors of physical therapists. Phys Ther.

[6] Barnard S, Wiles R (2001). Evidence-based physiotherapy: Physiotherapists’ attitudes and experiences in the Wessex area. Physiotherapy.

[7] Bourne J, Dzledzic K, Morris S, Jones P, Sim J (2007). Survey of the perceived professional, educational and personal needs of physiotherapists in primary care and community settings. Health Soc Care Commun.

[8] Harding KE, Porter J, Horne-Thompson A, Donley E, Taylor NF (2014). Not enough time or a low priority? Barriers to evidence-based practice for allied health clinicians. J Contin Educ Heal Prof.

[9] Metcalfe C, Lewin R, Wisher S, Perry S, Bannigan K, Moffett JK (2001). Barriers to implementing the evidence base in four NHS therapies. Physiotherapy.

[10] Stander J, Grimmer K, Brink Y (2021). Time as a barrier to evidence uptake-a qualitative exploration of the concept of time for clinical practice guideline uptake by physiotherapists. J Eval Clin Pract.

[11] Hannes K, Staes F, Goedhuys J, Aertgeerts B (2009). Obstacles to the implementation of evidence-based physiotherapy in practice: a focus group-based study in Belgium (Flanders). Physiother Theory Pract.

[12] Macleod MR, Michie S, Roberts I (2014). Biomedical research: increasing value, reducing waste. Lancet.

[13] Ioannidis JP (2016). Why Most clinical research is not useful. PLoS Med.

[14] Chalmers I, Glasziou P (2009). Avoidable waste in the production and reporting of research evidence. Lancet.

[15] Chalmers I, Essali A, Rezk E, Crowe S (2012). Is academia meeting the needs of non-academic users of the results of research?. Lancet.

[16] McCambridge AB, Nasser AM, Mehta P, Stubbs PW, Verhagen AP (2021). Has reporting on physical therapy interventions improved in 2 decades? An analysis of 140 trials reporting on 225 interventions. J Orthop Sports Phys Ther.

[17] Davidson SRE, Kamper SJ, Haskins R (2021). Exercise interventions for low back pain are poorly reported: a systematic review. J Clin Epidemiol..

[18] Concannon TW, Meissner P, Grunbaum JA (2012). A new taxonomy for stakeholder engagement in patient-centered outcomes research. J Gen Intern Med.

[19] Glasgow RE, Vinson C, Chambers D, Khoury MJ, Kaplan RM, Hunter C (2012). National Institutes of Health approaches to dissemination and implementation science: current and future directions. Am J Public Health.

[20] Atkins L, Francis J, Islam R (2017). A guide to using the theoretical domains framework of behaviour change to investigate implementation problems. Implement Sci.

[21] Davis R, Campbell R, Hildon Z, Hobbs L, Michie S (2015). Theories of behaviour and behaviour change across the social and behavioural sciences: a scoping review. Health Psychol Rev.

[22] Michie S (2005). Making psychological theory useful for implementing evidence-based practice: a consensus approach. Qual Saf Health Care.

[23] Squires JE, Graham I, Bashir K (2019). Understanding context: a concept analysis. J Adv Nurs.

[24] Presseau J, McCleary N, Lorencatto F, Patey AM, Grimshaw JM, Francis JJ (2019). Action, actor, context, target, time (AACTT): a framework for specifying behaviour. Implement Sci.

[25] Glanz K, Bishop DB (2010). The role of behavioral science theory in development and implementation of public health interventions. Annu Rev Public Health.

[26] Zadro JR, O'Keeffe M, Allison JL, Lembke KA, Forbes JL, Maher CG (2020). Effectiveness of implementation strategies to improve adherence of physical therapist treatment choices to clinical practice guidelines for musculoskeletal conditions: systematic review. Phys Ther.

[27] Fairbrother G, Cashin A, Conway MR, Symes MA, Graham I (2016). Evidence based nursing and midwifery practice in a regional Australian healthcare setting: Behaviours, skills and barriers. Collegian.

[28] Ducat WH, Kumar S (2015). A systematic review of professional supervision experiences and effects for allied health practitioners working in non-metropolitan health care settings. J Multidiscip Healthc..

[29] Cane J, O’Connor D, Michie S (2012). Validation of the theoretical domains framework for use in behaviour change and implementation research. Implement Sci.

[30] Thomas S, Mackintosh S (2014). Use of the theoretical domains framework to develop an intervention to improve physical therapist management of the risk of falls after discharge. Phys Ther.

[31] Packer-Muti B (2010). Conducting a focus group. Qual Report.

[32] Willis K, Green J, Daly J, Williamson L, Bandyopadhyay M (2009). Perils and possibilities: achieving best evidence from focus groups in public health research. Aust N Z J Public Health.

[33] Karnieli-Miller O, Strier R, Pessach L (2008). Power relations in qualitative research. Qual Health Res.

[34] Meyer ML, Louder CN, Nicolas G (2022). Creating with, not for people: theory of change and logic models for culturally responsive community-based intervention. Am Educ Res J..

[35] National Health and Medical Research Council (NHMRC) (2018). Australian code for responsible conduct of research Canberra.

[36] Australian Bureau of Statistics. Australian statistical geography standard (ASGS) Edition 3. https://www.abs.gov.au/statistics/standards/australian-statistical-geography-standard-asgs-edition-3/jul2021-jun2026. Published 20 July 2021. Accessed 22 Aug 2022.

[37] Onwuegbuzie AJ, Leech NL (2007). Sampling designs in qualitative research: making the sampling process more public. Qual Rep..

[38] Physiotherapy Evidence Database (PEDro). Codes. https://pedro.org.au/english/learn/indexing-criteria-and-codes/#part-2. Published 2021. Updated 15th October 2021. Accessed 14 Oct 2021.

[39] Turner DW (2010). Qualitative interview design: a practical guide for novice investigators. Qual Rep..

[40] Project Redcap. REDcap. https://www.project-redcap.org/. Published 2021. Updated 14 Oct 2021. Accessed 14 Oct 2021.

[41] Zoom. Zoom. https://zoom.us/. Published 2021. Updated 14th October 2021. Accessed 14th October 2021.

[42] International Q. NVivo. https://www.qsrinternational.com/nvivo-qualitative-data-analysis-software/home. Published 2021. Updated 14 Oct 2021. Accessed 14 Oct 2021.

[43] Fereday J, Muir-Cochrane E (2006). Demonstrating rigor using thematic analysis: a hybrid approach of inductive and deductive coding and theme development. Int J Qual Methods.

[44] Gale NK, Heath G, Cameron E, Rashid S, Redwood S (2013). Using the framework method for the analysis of qualitative data in multi-disciplinary health research. BMC Med Res Methodol.

[45] O’Cathain A, Murphy E, Nicholl J (2010). Three techniques for integrating data in mixed methods studies. BMJ.

[46] Hennink MMHIBA (2020). Qualitative research methods.

[47] Braun V, Clarke V (2019). Reflecting on reflexive thematic analysis. Qual Res Sport Exerc Health.

[48] Bernard HR, Ryan GW (2010). Analyzing qualitative data: systematic approaches.

[49] Foster RL (1997). Addressing epistemologic and practical issues in multimethod research: a procedure for conceptual triangulation. ANS Adv Nurs Sci.

[50] Mathison S (1988). Why triangulate?. Educ Res.

[51] da Silva TM, Costa Lda C, Garcia AN, Costa LO (2015). What do physical therapists think about evidence-based practice? A systematic review. Man Ther.

[52] Grimmer-Somers K, Lekkas P, Nyland L, Young A, Kumar S (2007). Perspectives on research evidence and clinical practice: a survey of Australian physiotherapists. Physiother Res Int.

[53] Iles R, Davidson M (2006). Evidence based practice: a survey of physiotherapists’ current practice. Physiother Res Int.

[54] Lizarondo LM, Grimmer-Somers K, Kumar S, Crockett A (2012). Does journal club membership improve research evidence uptake in different allied health disciplines: a pre-post study. BMC Res Notes.

[55] Fruth SJ, Van Veld RD, Despos CA, Martin RD, Hecker A, Sincroft EE (2010). The influence of a topic-specific, research-based presentation on physical therapists’ beliefs and practices regarding evidence-based practice. Physiother Theory Pract.

[56] Sackett DL, Rosenberg WMC, Gray JAM, Haynes RB, Richardson WS (1996). Evidence based medicine: what it is and what it isn't. BMJ.

[57] Herbert RD, Sherrington C, Maher C, Moseley AM (2001). Evidence-based practice -- imperfect but necessary. Physiother Theory Pract.

[58] Kamper SJ, Haanstra TM, Simmons K (2018). What do patients with chronic spinal pain expect from their physiotherapist?. Physiother Can.

[59] Licina P, Johnston M, Ewing L, Pearcy M (2013). Patient expectations, outcomes and satisfaction: related, relevant or redundant?. Evid-Based Spine-Care J.

[60] Goossens ME, Vlaeyen JW, Hidding A, Kole-Snijders A, Evers SM (2005). Treatment expectancy affects the outcome of cognitive-behavioral interventions in chronic pain. Clin J Pain.

[61] Hush JM, Cameron K, Mackey M (2011). Patient satisfaction with musculoskeletal physical therapy care: a systematic review. Phys Ther.

[62] Bowling A, Rowe G, Lambert N (2012). The measurement of patients' expectations for health care: a review and psychometric testing of a measure of patients' expectations. Health Technol Assess..

[63] Haanstra TM, Hanson L, Evans R (2013). How do low back pain patients conceptualize their expectations regarding treatment? Content analysis of interviews. Eur Spine J.

[64] Unertl KM, Fair AM, Favours JS, Dolor RJ, Smoot D, Wilkins CH (2018). Clinicians’ perspectives on and interest in participating in a clinical data research network across the southeastern United States. BMC Health Serv Res.

[65] Chalmers I, Atkinson P, Badenoch D (2019). The James Lind initiative: books, websites and databases to promote critical thinking about treatment claims, 2003 to 2018. Res Involv Engagem.

[66] National Health and Medical Research Council (NHMRC). Statement on consumer and community involvement in health and medical research, consumers forum of Australia. Canberra: National Health and Medical Research Council (NHMRC); 2016.

[67] Graham ID, McCutcheon C, Kothari A (2019). Exploring the frontiers of research co-production: the integrated knowledge translation research network concept papers. Health Res Policy Syst.

[68] Ostrom E (1999). Crossing the great divide: coproduction, synergy, and development. World Dev.

