# Supplementary material for: Physiotherapists’ opinions, barriers, and enablers to providing evidence-based care: a mixed-methods study
Source: BMC Health Serv Res. 2022 Nov 21;22:1382. doi: 10.1186/s12913-022-08741-5 (PMC9677623; doi:10.1186/s12913-022-08741-5)
Supplement: Supplementary file 1 — Additional file 1: Appendix 1. [file 12913_2022_8741_MOESM1_ESM.docx]

# Appendix 1.

**Survey results**

49 participants answered the survey. 48 participants completed the full survey. One participant completed only up to question 21 (Please tell us how much you agree with the following statements, “It’s hard to apply research evidence because.” - ‘A lot of research doesn’t answer my clinical problems.”). A subgroup analysis of survey responses from regional, rural, and remote clinical practice regions can be found as online supplement 3.

*Demographics*

| ***Table 1:*** *Demographics characteristics* | |
| --- | --- |
| **N** | 49 |
| **Age (years)** | 38 (27 - 49) |
| **Years in practice**  1-5  6-10  11-15  16-20  21+ | 10 (20%)  16 (33%)  6 (12%)  6 (12%)  11 (23%) |
| **Location of practice**  Regional  Rural  Remote  Don’t know  I practice outside of Australia | 35 (72%)  3 (6%)  1 (2%)  1 (2%)  9 (18%) |
| **Clinical area of practice**  Cardiothoracics  Chronic Pain  Chronic respiratory disease  Continence and Women’s Health  Gerontology  Musculoskeletal  Neurology  Oncology  Orthopaedics  Sports  Whiplash | 1 (2%)  5 (10%)  1 (2%)  1 (2%)  1 (2%)  29 (60%)  0 (0%)  1 (2%)  3 (6%)  6 (12%)  1 (2%) |

*The opinions about evidence*

| ***Table 2:*** *Clinicians’ opinions to research evidence* | | | | | |
| --- | --- | --- | --- | --- | --- |
| **‘Research evidence informs my treatment choices’***  **Strongly disagree**  **Disagree**  **Neutral**  **Agree**  **Strongly agree** | 3/48(6%)  1/48(2%)  3/48(6%)  18/48(38%)  23/48(48%) | | | | |
| **‘How important are the following factors when making clinical decisions’**  **Patient expectations** | ***Unimportant*** | ***Slightly important*** | ***Moderately important*** | ***Important*** | ***Very important*** |
|  | 0/49(0%) | 2/49(4%) | 3/49(6%) | 15/49(31%) | 29/49(59%) |
| **Colleagues’ choices** | 11/49(23%) | 20/49(41%) | 9/49(18%) | 9/49(18%) | 0/49(0.0%) |
| **Evidence** | 0/49(0.0%) | 0/49(0%) | 2/49(4%) | 26/49(53%) | 21/49(43%) |

*One incomplete value - question not a forced response.

*Describing research access*

| ***Table 3:*** *How do clinicians access research (n=49)* | | | | | |
| --- | --- | --- | --- | --- | --- |
| **‘How much time do you spend accessing research evidence in the below mediums:’**  Research summaries | **less than 10 minutes per week** | **10-30 minutes per week** | **30 minutes – 1 hour per week** | **1-2 hours per week** | **over 2 hours per week** |
|  | 8 (16%) | 18 (37%) | 8 (16%) | 11 (23%) | 4 (8%) |
| Listening to podcasts | 18 (37%) | 10 (20%) | 9 (19%) | 7 (14%) | 5 (10%) |
| Research article abstracts | 12 (25%) | 21 (43%) | 10 (20%) | 2 (4%) | 4 (8%) |
| Research full text articles | 14 (29%) | 15 (31%) | 7 (14%) | 6 (12%) | 7 (14%) |
| Reading blogs | 13 (27%) | 20 (41%) | 8 (16%) | 6 (12%) | 2 (4%) |

*Barriers to research evidence access and application*

| ***Table 4:*** *Barriers to evidence access and application* | | | | | |
| --- | --- | --- | --- | --- | --- |
| **‘It’s hard to apply evidence because’**  ***Knowledge:*** I don’t know where to find research evidence | **Strongly disagree** | **Disagree** | **Undecided** | **Agree** | **Strongly agree** |
|  | 22/49(45%) | 19/49(39%) | 4/49(8%) | 4/49(8%) | 0/49(0%) |
| ***Skills***: It’s hard to access research evidence | 12/49(24%) | 15/49(31%) | 4/49(8%) | 14/49(29%) | 4/49(8%) |
| ***Skills***: It’s hard to know what good quality research evidence is | 8/49(16%) | 17/49(35%) | 9/49(18%) | 15/49(31%) | 0/49(0%) |
| ***Research relevance:*** A lot of research doesn’t answer my clinical problems | 3/49(6%) | 11/49(23%) | 8/49(16%) | 23/49(47%) | 4/49(8%) |
| ***Social norms:*** My clinic doesn’t value research evidence | 25/48(52%) | 13/48(27%) | 6/48(13%) | 4/48(8%) | 0/48(0%) |
| ***Perceived behavioural control:*** My patients expect certain treatments that aren’t evidence based | 2/48(4%) | 10/48(21%) | 7/48(15%) | 25/48(52%) | 4/48(8%) |
| ***Self-confidence:*** I am not confident to provide evidence-based treatments | 22/48(46%) | 22/48(46%) | 2/48(4%) | 1/48(2%) | 1/48(2%) |
| ***Resources:*** I don’t have the equipment to implement treatments that are evidence based | 22/48(46%) | 18/48(38%) | 3/48(6%) | 4/48(8%) | 1/48(2%) |
| ***Resources (time):*** I don’t have the time to implement treatments described in research | 18/48(38%) | 22/48(46%) | 6/48(12%) | 2/48(4%) | 0/48(0%) |
| ***Routine/habit:*** I have found it too hard to get into the habit of accessing and applying research evidence | 19/48(40%) | 16/48(33%) | 7/48(15%) | 3/48(6%) | 3/48(6%) |
| ***Stress:*** All things considered, trying to implement evidence-based treatments causes too much stress on my work | 15/48(32%) | 25/48(52%) | 4/48(8%) | 4/48(8%) | 0/48(0%) |
| ***Motivation:*** It’s hard to be motivated to routinely incorporate evidence into my practice | 18/48(38%) | 20/48(42%) | 5/48(10%) | 4/48(8%) | 1/48(2%) |

*Enablers to evidence application*

| ***Table 5:*** *Potential enablers to evidence application (n=48)* | | | | | |
| --- | --- | --- | --- | --- | --- |
| **‘It would make it easier to apply evidence if’**  ***Training:*** I get further training in critical appraisal of research | **Strongly disagree** | **Disagree** | **Undecided** | **Agree** | **Strongly agree** |
|  | 4 (8%) | 5 (10%) | 7 (15%) | 25 (52%) | 7 (15%) |
| ***Research Question design***: I get support to answer questions relevant my patients/clinic | 2 (4%) | 5 (10%) | 4 (8%) | 32 (67%) | 5 (11%) |
| ***Research generation***: I become involved in doing good research | 4 (8%) | 8 (17%) | 6 (12%) | 21 (44%) | 9 (19%) |
| ***Mentorship, connection, reflection:*** I were able to connect with other like-minded clinicians to discuss applying evidence in practice | 1 (2%) | 3 (6%) | 3 (6%) | 24 (50%) | 17 (36%) |

*Network specifics*

*‘If we were to set up a regional physio network, how would this network support your work?’*

| ***Table 6:*** *Specific aspects of a physiotherapy research network that would support evidence-based practice (n=48)* | |
| --- | --- |
| **Specific function** | **Number of participants in support n/N (%)** |
| Networking events (6 monthly) | 32 (67%) |
| Physiotherapy specific professional development | 33 (69%) |
| Shared professional development | 38 (79%) |
| Access to online discussion forums (to discuss clinical cases and ideas) | 25 (52%) |
| Access to private social media discussion group | 27 (56%) |
