# Supplementary material for: Physiotherapists’ opinions, barriers, and enablers to providing evidence-based care: a mixed-methods study
Source: BMC Health Serv Res. 2022 Nov 21;22:1382. doi: 10.1186/s12913-022-08741-5 (PMC9677623; doi:10.1186/s12913-022-08741-5)
Supplement: Supplementary file 2 — Additional file 2. Online supplement 1. [file 12913_2022_8741_MOESM2_ESM.docx]

# Online supplement 1

Interview schedule

| Section I. How do we improve practice  We understand a lot of our patients have a rough ride through the healthcare system and there are many factors involved in providing ‘high value’ care.   1. **How do we improve practice?**   Section II. Opinion on research  **2) Where does research fit into this?**  Probe (if needed) - What is the purpose of research?  Section III. Problems  **3) What type of issues do you think this network could help with?**  Section IV. Clinician’s solutions  **4) In your eyes, how do you see this network being successful?**  Probes - What would you need? Time? Resources? Face to face/online?  Section V. **‘other’ problems** and whether network is the right place to address them  Let’s step away for a while from the idea of the practice-based network. In our earlier discussions with physios, we’ve heard about problems that clinicians face related to running their business, or those related to communication issues with other professionals, i.e., doctors. Non-clinical problems.  **5) Is that something that you experience as well?**  6) (if yes) Can you think of other ways to address these problems? (They don’t have to involve a practice-based research network)  (If no- don’t go any further)  Would practice-based network be helpful in addressing these problems at all? If yes, why/how? If no, why not? What else would help you address these problems? |
| --- |
