# Supplementary material for: Physiotherapists’ opinions, barriers, and enablers to providing evidence-based care: a mixed-methods study
Source: BMC Health Serv Res. 2022 Nov 21;22:1382. doi: 10.1186/s12913-022-08741-5 (PMC9677623; doi:10.1186/s12913-022-08741-5)
Supplement: Supplementary file 3 — Additional file 3. Online supplement 2. [file 12913_2022_8741_MOESM3_ESM.docx]

# Online supplement 2

# Final theme matrix

***Research Question 1:*** Understanding the opinions of regional physiotherapists towards research evidence in relation to their clinical decision making

| Theme | Subtheme | Quant | Qual | Agreement | Partial agreement | Dissonance | TDF |
| --- | --- | --- | --- | --- | --- | --- | --- |
| Positive attitude |  | Y | Y | Y |  |  |  |
| Integration of many factors | Patient expectations | Y | Y | Y |  |  | Beliefs about capabilities (perceived behavioural control), Social influences, Context and resources |
|  | Colleagues | Y | Y | N |  | Y |  |
|  | Clinical environment | Silence | Y |  | Y - Only Qual |  |  |
| Tension between factors involved in clinical decisions. | *RE versus experience* | Silence | Y |  | Y |  |  |
|  | *Business demands versus evidence-based practice* | Silence | Y |  | Y |  |  |
|  | *Patient expectations versus evidence informed treatment* | Silence | Y |  | Y |  |  |

***Research Question 2:*** How do regional physiotherapists access research evidence?

| Theme | Subtheme | Quant | Qual | Agreement | Partial agreement | Dissonance |
| --- | --- | --- | --- | --- | --- | --- |
| Clinicians dedicate a small amount of time consuming research evidence, across multiple mediums |  | Y | Silence |  | Y |  |
| Triaging research evidence | Unclear how physiotherapists assess the quality of RE. | Silence | Y |  | Y |  |
|  | Triaging or opinion forming may not be based on the content of research evidence or article content | Silence | Y |  | Y |  |

***Research question 3:*** Understanding the barriers and enablers of regional physiotherapists in evidence access

| TDF Domain (construct) | Quant | Qual | Agreement | Partial agreement | Dissonance | Theme |
| --- | --- | --- | --- | --- | --- | --- |
| Knowledge | ↓ Not barrier | ↓ Not barrier | Y |  |  | **Prioritisation** |
| Skills | ↓ Not barrier | Not barrier | Y |  |  |  |
| Social/professional role/ID | - | - |  |  |  |  |
| Beliefs about capabilities | - | - |  |  |  |  |
| Beliefs about consequences | - | - |  |  |  |  |
| Motivation and goals | - | - |  |  |  |  |
| Memory, attention and decision making | - | - |  |  |  |  |
| Environmental context and resources (time) | ↓Not a barrier | ↑Barrier (survey and focus group) |  |  | Y (Across quant & qual) | **Prioritisation** |
| Environmental context and resources (paywalls) | - | ↑ Barrier and Enabler (survey) | Y |  |  | **Paywalls** |
| Social influences | ↑ Enabler | ↑Barrier |  |  | Y |  |
| Emotion | - | - |  |  |  |  |
| Behavioural regulation | - | - |  |  |  |  |
| Nature of behaviours | - | - |  |  |  |  |
|  |  |  |  |  |  |  |
|  |  |  |  |  |  |  |
|  |  |  |  |  |  |  |
|  |  |  |  |  |  |  |
|  | Skills | RE volume |  | Y |  |  |
|  |  | RE uncertainty |  | Y |  |  |
|  | Perceived behavioural control | Patient expectations | Y |  |  |  |
|  |  | Therapeutic Alliance |  | Y |  |  |
|  | System support | Lack of general system support |  | Y |  |  |
|  |  | Lack of culture of accountability |  | Y |  |  |
|  |  | Lack of incentives |  | Y |  |  |

***Research Question 4:*** Understanding the barriers and enablers of regional physiotherapists in evidence application.

| TDF Domain (construct) | Quant | Qual | Agreement | Partial agreement | Dissonance | Theme |
| --- | --- | --- | --- | --- | --- | --- |
| Knowledge | - | - |  |  |  |  |
| Skills | ↑Barrier | ↑Barrier | Y |  |  | **Volume of evidence** |
| Social/professional role/ID | - | - |  |  |  |  |
| Beliefs about capabilities | ↑Barrier | ↑Barrier | Y |  |  | **Patient expectations** |
| Beliefs about capabilities (confidence) | ↓Not barrier |  |  |  |  |  |
| Beliefs about consequences | - | ↑Barrier |  | Y |  |  |
| Motivation and goals | ↓Not barrier | - |  |  |  |  |
| Memory, attention and decision making | - | - |  |  |  |  |
| Environmental context and resources (equipment) | ↓Not barrier | - |  |  |  |  |
| Environmental context and resources (time) | ↓Not barrier | - |  |  |  |  |
| Environmental context and resources | - | ↑Barrier and Enabler |  |  |  | **System Factors** |
| Social influences (norms | ↓Not barrier | ↑Barrier and Enabler | Y |  | Y | **Mentorship**  **Culture of accountability** |
| Emotion | ↓Not barrier | - |  |  |  |  |
| Behavioural regulation | - | - |  |  |  |  |
| Nature of behaviours | ↓Not barrier | - |  |  |  |  |
| OTHER |  |  |  |  |  | **Research Relevance** |

Mixed methods results for research objective 1: *Understanding the opinions of regional physiotherapists towards evidence in relation to their clinical decision making (n=57).*

| **TDF Domain (construct)** | **Quantitative data (survey)** | **Qualitative data (focus group)** | **Theme** |
| --- | --- | --- | --- |
| N/A | - 86% either agreed or strongly agreed that ‘evidence informs my treatment choices.’ Nearly ½ half of the total sample strongly agreed. - 96% of participants consider evidence as either important or very important when making clinical decisions. | *“I think evidence is kind of crucial to pushing the profession forward.”* | ***Positive attitude.***  Regional physiotherapists have a positive attitude towards evidence. |
| Beliefs about capabilities (perceived behavioural control) | **Patient expectations**   - 90% of participants considered patient expectations as either important or very important when making clinical decisions. 59% of the total sample considered patient expectations as very important. | **Patient expectations**  *“Before we can start to apply things such as the best research and best evidence. Being able to get to that next step of providing our services, I think you’ve got to have the patient on board and part of that journey with you.”* | ***Integration of many factors.*** Clinician’s report having to consider many other factors when using evidence in clinical decision making; patient expectations, colleagues’ choices, and the clinical environment. |
| Social influences | **Colleagues’ choices**   - 77% of participants considered ‘colleagues’ choices’ as either slightly important (41%), moderately important (18%), or important (18%). 23% of participants rated ‘colleagues’ choices’ as unimportant. |  |  |
| Context and resources (Environmental constraints) |  | **Clinical environment**  *“So, it comes back to constraints - either environmental or physical constraints, or constraints what they can do or how the business is set up.”* |  |
| N/A |  | **Clinical experience**  *“Trying to keep pace with what is shown in the literature to be effective versus not effective, and yet still trying to balance an experiential process where you know that for some conditions… And constantly trying to balance that has always been a bit of a wrestle.”* | ***Tension between factors.***  Clinicians report conflict between evidence and other considerations of clinical practice when making clinical decisions: like clinical experience, business demands, and patient expectations. |
|  |  | **Business demands**  *“As far as delivering high quality care, I have always struggled with that one, I’ve worked in private. I feel like it’s really hard to give a good treatment in a short period of time that’s financially viable that a person is willing to pay for.”* |  |
|  |  | **Patient expectations**  *“The patient can often be very confused around what to believe or pursue for treatment. So often trying to bring that back into some sort of order and structure for creating a management plan for them can be a bit challenging.”* |  |

Mixed methods results for research objective 2: *How do regional physiotherapists access evidence? (n=57).*

| **Quantitative data (survey)** | **Qualitative data (focus group)** | **Theme** |
| --- | --- | --- |
| - Over 50% of participants reported spending 30 minutes or less per week on accessing evidence, across all mediums. - 53% of participants spent 30 minutes or less accessing evidence summaries. 23% spent 1 - 2 hours per week accessing evidence summaries. - 57% of participants spent 30 minutes or less per week accessing evidence in podcast format. - 68% of participants spent 30 minutes or less per week accessing article abstracts. - 60% of participants spent 30 minutes or less per week accessing full text articles. - 68% of participants spent 30 minutes or less per week accessing blogs that directly discuss evidence. |  | ***Clinicians dedicate a small amount of time accessing evidence, over multiple mediums.*** |
|  | *“So yeah, if any physio related stuff comes up [on Facebook], I’ll usually notice it and triage if I’m actually going to read it or not.”*  *“But a few times when I’ve gone in there to try to have a look and read the abstract, then I sort of think, ‘ah that’s worth a read.”* | ***Triaging evidence.***  Sources of evidence (particularly on social media) is triaged.  Interesting, or attention grabbing, evidence is prioritised. |

# Subgroup analysis of survey responses from regional, rural, and remote clinical practice regions

*Demographics*

| ***Table 1:*** *Participant characteristics (regional subgroup)* | |
| --- | --- |
| **N** | 35 |
| **Age (years)** | 37 (30 - 46) |
| **Years in practice**  1-5  6-10  11-15  16-20  21+ | 7 (20%)  11 (32%)  5 (14%)  5 (14%)  7 (20%) |
| **Location of practice**  Regional | 35 (100%) |
| **Clinical area of practice**  Cardiothoracics  Chronic Pain  Chronic respiratory disease  Continence and Women’s Health  Gerontology  Musculoskeletal  Neurology  Oncology  Orthopaedics  Sports  Whiplash | 0 (0%)  2 (6%)  1 (3%)  0 (0%)  1 (3%)  22 (62%)  0 (0%)  0 (0%)  3 (9%)  5 (14%)  1 (3%) |

*The opinions about evidence*

| ***Table 2:*** *Clinicians’ opinions to research evidence (n=35)* | | | | | |
| --- | --- | --- | --- | --- | --- |
| **‘Research evidence informs my treatment choices’***  **Strongly disagree**  **Disagree**  **Neutral**  **Agree**  **Strongly agree** | 3 (8%)  0 (0%)  1 (6%)  16 (41%)  14 (45%) | | | | |
| **‘How important are the following factors when making clinical decisions’**  **Patient expectations** | ***Unimportant*** | ***Slightly important*** | ***Moderately important*** | ***Important*** | ***Very important*** |
|  | 0 (0%) | 2 (6%) | 2 (6%) | 10 (28%) | 21 (60%) |
| **Colleagues choices** | 6 (17%) | 15 (43%) | 7 (20%) | 7 (20%) | 0 (0.0%) |
| **Evidence** | 0 (0.0%) | 0 (0%) | 1 (3%) | 20 (57%) | 14 (40%) |

*Describing research access*

| ***Table 3:*** *How do clinicians access research (n=35)* | | | | | |
| --- | --- | --- | --- | --- | --- |
| **‘How much time do you spend consuming research evidence in the below mediums:’**  Research summaries | **less than 10 minutes per week** | **10-30 minutes per week** | **30 minutes – 1 hour per week** | **1-2 hours per week** | **over 2 hours per week** |
|  | 5 (14%) | 14 (40%) | 6 (17%) | 10 (29%) | 0 (0%) |
| Listening to podcasts | 11 (32%) | 8 (23%) | 7 (20%) | 5 (14%) | 4 (11%) |
| Research article abstracts | 8 (23%) | 17 (49%) | 8 (22%) | 1 (3%) | 1 (3%) |
| Research full text articles | 11 (32%) | 14 (40%) | 5 (14%) | 4 (11%) | 1 (3%) |
| Reading blogs | 11 (32%) | 14 (40%) | 6 (17%) | 4 (11%) | 0 (0%) |

*Barriers to research evidence access and application*

| ***Table 4:*** *Barriers to evidence access and application* | | | | | | |
| --- | --- | --- | --- | --- | --- | --- |
| **TDF Domain (construct)** | **‘It’s hard to apply evidence because’**  ***a)*** I don’t know where to find research evidence | **Strongly disagree** | **Disagree** | **Undecided** | **Agree** | **Strongly agree** |
| ***Knowledge*** |  | 13/35(37%) | 15/35(43%) | 4/35(11%) | 3/35(9%) | 0/35(0%) |
| ***Skills*** | ***b)*** It’s hard to access research evidence | 8/35(23%) | 10/35(29%) | 3/35(8%) | 13/35(37%) | 1/35(3%) |
| ***Skills*** | ***c)*** It’s hard to know what good quality research evidence is | 8/35(23%) | 11/35(31%) | 7/35(20%) | 9/35(26%) | 0/35(0%) |
| ***-*** | ***d)*** A lot of research doesn’t answer my clinical problems* | 3/35(9%) | 5/35(14%) | 6/35(17%) | 20/35(57%) | 1/35(3%) |
| ***Social influences (social norms)*** | ***e)*** My clinic doesn’t value research evidence | 20/34(59%) | 7/34(20%) | 5/34(15%) | 2/34(6%) | 0/34(0%) |
| ***Beliefs about capabilities (perceived behavioural control)*** | ***f)*** My patients expect certain treatments that aren’t evidence based | 2/34(6%) | 4/34(12%) | 7/34(20%) | 20/34(59%) | 1/34(3%) |
| ***Beliefs about capabilities (self-confidence)*** | ***g)*** I am not confident to provide evidence based treatments | 17/34(50%) | 15/34(44%) | 2/34(6%) | 0/34(0%) | 0/34(0%) |
| ***Environmental context and resources (Resources - equipment)*** | ***h)*** I don’t have the equipment to implement treatments that are evidence based | 16/34(47%) | 12/34(35%) | 3/34(9%) | 2/34(6%) | 1/34(3%) |
| ***Environmental context and resources (time)*** | ***i)*** I don’t have the time to implement treatments described in research | 13/34(38%) | 15/34(44%) | 5/34(15%) | 1/34(3%) | 0/34(0%) |
| ***Nature of the behaviour (routine/automatic/habit)*** | ***j)*** I have found it too hard to get into the habit of accessing and applying research evidence | 14/34(41%) | 12/34(35%) | 5/34(15%) | 2/34(6%) | 1/34(3%) |
| ***Emotion (stress)*** | ***k)*** All things considered, trying to implement evidence-based treatments causes too much stress on my work | 11/34(32%) | 19/34(56%) | 3/34(9%) | 1/34(3%) | 0/34(0%) |
| ***Motivation and goals*** | ***l)*** It’s hard to be motivated to routinely incorporate evidence into my practice | 13/34(38%) | 15/34(44%) | 4/34(12%) | 1/34(3%) | 1/34(3%) |

*One participant only answered up to question *d) A lot of research doesn’t answer my clinical problems*

*Enablers to evidence application*

| ***Table 5:*** *Potential enablers to evidence application (n=34)* | | | | | | |
| --- | --- | --- | --- | --- | --- | --- |
| **TDF Domain (construct)** | **‘It would make it easier to apply evidence if’**  ***Training:*** I get further training in critical appraisal of research | **Strongly disagree** | **Disagree** | **Undecided** | **Agree** | **Strongly agree** |
| *-* |  | 2 (8%) | 4 (10%) | 7 (19%) | 17 (53%) | 4 (10%) |
| ***-*** | ***Research Question design***: I get support to answer questions relevant my patients/clinic | 1 (0%) | 3 (10%) | 3 (8%) | 22 (66%) | 5 (13%) |
| ***-*** | ***Research generation***: I become involved in doing good research | 3 (8%) | 6 (18%) | 5 (16%) | 15 (45%) | 5 (13%) |
| ***Social influences (social norms)*** | ***Mentorship:*** I were able to connect with other like-minded clinicians to discuss applying evidence in practice | 0 (0%) | 2 (8%) | 2 (5%) | 17 (53%) | 13 (34%) |

*Network specifics*

*‘If we were to set up a regional physio network, how would this network support your work?’*

| ***Table 6:*** *Specific aspects of a physiotherapy research network that would support evidence-based practice (n=34)* | |
| --- | --- |
| **Specific function** | **Number of participants in support** |
| Networking events (6 monthly) | 26 (76%) |
| Physiotherapy specific professional development | 27 (79%) |
| Shared professional development | 30 (88%) |
| Access to online discussion forums (to discuss clinical cases and ideas) | 20 (59%) |
| Access to private social media discussion group | 22 (65%) |
